# Pornography. The politics of legal changes. An opinion article

**DOI:** 10.3389/fsoc.2023.1250012

**Published:** 2023-11-27

**Authors:** Håkan Hydén

**Affiliations:** ^1^Academy for Learning, Humanities and Society, Halmstad University, Halmstad, Sweden; ^2^Department of Sociology of Law, Lund University, Lund, Sweden

**Keywords:** pornography, sex purchase law, comparative law, legal strategies for political change, victim less crime

## 1 Pornography as something harmful

Using statistical data, Waltman (2021) describes how pornography as a social practice disregards equality by exploiting people from multiple disadvantaged backgrounds and contributes to gender-based violence. The exploitation and abuse that pornography production inflicts on members of disadvantaged groups is connected to sexual aggression and gender-based violence. Pornography has a close link to prostitution. There is an association between increased pornography consumption and increased sexual aggression, increases in attitudes promoting violence against women, and more frequent purchases of prostituted sex.

Robust and statistically significant data show that consumption of pornography contributes to substantially more gender-based violence and to an array of attitudes that minimize, trivialize, or normalize it (Waltman, 2021). Studies confirm strong links between childhood abuse and neglect, homelessness, and prostitution. Poverty is a predominant reason for entering the sex industry. The pornography industry can use money to coerce prostitutes to participate in unsafe and borderline cruel practices.

Waltman raises the question: What conditions obstruct and enable legal challenges to the production and consumption harms of pornography, and what are the alternatives? He claims that examination of the politics of legal challenges to pornography may provide a blueprint to guide studies of other complicated, seemingly intractable, social problems. This includes challenges to climate change that face “problematic existing norms” that obstruct efforts to mitigate the emission of greenhouse gases in the atmosphere. Waltman recommends a problem-driven approach following the political theorist (Shapiros, 2002).^1^

### 1.1 Theory and method

In order to build a theory that makes legal challenges to exploitative practices more successful generally, and particularly with respect to pornography, Waltman uses a theory he calls hierarchy theory. This is derived in part from the scholarship of Catherine MacKinnon, who frequently uses the term “hierarchy” to describe the problem her feminist theory addresses (MacKinnon, 2016). This theory, Waltman asserts, can be tested by combining qualitative within-case methods that include pattern matching cases from the United States, Canada, and Sweden. To the extent that institutionalizing the perspectives and political and legal interests of subordinated groups, such as prostitution survivors and women in abusive relationships, is likely to result in progressive policy change, it supports hierarchy theory.

The study conducted employs the comparative case study method to explain what obstructs or enables legal challenges to pornography's harms. Waltman argues for an analytical generalization rather than a statistical generalization. He refers to pattern matching, also called the congruence method, as a useful method for testing the explanatory power of theories, including the hierarchy theory (George and Bennett, 2004). It tests whether data conforms to theoretically predicted patterns (Lange, 2013). The study adopts the most similar systems design as a means of strengthening the within-case findings from pattern matching across units. The United States, Canada, and Sweden are similar on several key measures of democracy, principally in recognizing the imperatives of sex equality, non-exploitation, and freedom of expression. The last-mentioned human right is the most used argument in favor of pornography.

### 1.2 Different legal strategies

The United States, Canada, and Sweden provide different legal strategies for combating gender-based violence. In the United States, there have been several legal approaches to challenge pornography. The influential Professor Catherine MacKinnon argued for a civil rights strategy. Pornography presents women as subordinate to men and is therefore a form of discrimination on basis of sex. This formed the content of the Minneapolis ordinance, which in 1984 was adopted by the preponderance of one voice in the City council. The ordinance provided remedies to those victimized by pornography. However, 2 years later, the U.S. Supreme Court affirmed a lower court ruling that invalidates the pornography ordinance approved in Indianapolis. The civil rights anti-pornography movements in Minneapolis and Indianapolis had gathered enough public momentum by 1985 to produce widespread national debate. As a result, a commission on pornography was tasked “to determine the nature, extent, and impact on society of pornography in the United States, and to make specific recommendations” (Waltman, 2021, p.216).

The Commission has what is likely the single most extensive investigation to date on the harms caused by the production and consumption of pornography. The Commission reached the conclusion that the pornography industry systematically violates human rights with apparent impunity. Similar anti- pornography ideas continued to generate interest among American politicians. Among other things, a Pornography Victims Protection Act was introduced. However, Congressional attempts to pass civil rights legislation against pornography was obstructed. The closest route to challenge the harms of pornography that remained was the obscenity law, despite not being victim-centered or concerned with sex discrimination. The problem remained, despite state prosecutions, and online pornography flourished. Waltman claims that the obscenity law facially protects no compelling or substantial interests regarding pornography's harms.

### 1.3 A discussion of efficiency and consequences

Still, the most direct legal tool available in Canada against pornography is the obscenity law. A special Committee on Pornography and Prostitution was set up in 1983 after an intense public pressure. Pornography was defined in a gender-neutral term and the law that followed became toothless. The anti-pornography group in Canada therefore used another strategy to intervene in criminal cases regarding obscenity. However, courts relied on contemporary standards of tolerance rather than more objective tools, whereby a normalization of sexual assault determined the assessment of harm. Waltman would rather have seen an anti- pornography civil rights law enabling those directly hurt by pornography, who have the strongest incentives, to initiate legal action on their own – with or without government assistance (Waltman, 2021, p.305).

Despite the proportion of female parliament members in Sweden in the first decade of the second millennium, almost 50%, no legal efforts were undertaken to challenge pornography. When it comes to prostitution, Sweden has criminalized those who pay for prostitution since 1999, not those selling. Sentences for buyers of sexual services range from fines to imprisonment for at most 1 year. Waltman concludes that, irrespective of the legality of prostitution, a symmetrical treatment of the parties involved would have been inconsistent with hierarchy theory. From the perspective of hierarchy theory, reducing the number of individuals in prostitution is imperative to the promotion of substantive equality. Swedish law has accomplished such a reduction when it comes to street prostitution.^2^ The legitimacy and thereby public support of the law has increased dramatically over the years (Waltman, 2021, p.346). There are indications that the level of prostitution in Sweden is reduced relative to that of other nations.

Swedish law is also unique in the sense that prostitution is, from a formal point of view, a victimless crime. This creates a problem since the judicial system seems to not (yet) regard prostituted people as victimized. Perpetrators of the law are not regarded as individually accountable for the harms afforded to those from whom they purchase sex. A discussion has taken place over whether those who engage in prostitution should pay taxes for the money they earn. However, this was rejected for two reasons. One is that it would be a public exploitation of those mired in prostitution and the second argument is that paying taxes implies permission to continue. Sex purchasing is regarded as a crime against the public order, not against people. The women are not seen as an injured party and thereby there are no consequences for the exploitation she experiences. This interpretation of the law was confirmed in a Supreme Court case in 2001. Prostitution was regarded as caused by structural factors without individual responsibility for either the selling women or the buyers. However, the Swedish government in 2011 clarified that prostitutes could be regarded as an injured party and claim damages, but that has then to be determined on a case-by-case basis. So far, no such successful civil lawsuit has been reported.

## 2 Conclusions

Some conclusions might be drawn from Waltman's study. The most obvious is that pornography contributes to gender inequality. He refers to almost 50 years of social science research showing that pornography fuels sexual aggression and attitudes supporting violence against women and the demand for prostitution. Consumers often wish to imitate pornography with reluctant partners. The main opposition to legal challenges to pornography is the conflict with freedom of expression. There are pros and cons from an empirical point of view based on the findings from different cases of legal strategies used in the respective countries.

Seen through the lens of hierarchy theory, obscenity laws were shown to be unreliable, vague, and vulnerable to misuse. In contrast to criminal obscenity laws, the empirical analysis of legal challenges indicates a stronger civil rights framework would amplify the perspectives, interests, and knowledge of survivors of the pornography industry and others harmed by it. Criminal obscenity law represents the perspectives of law enforcement and prosecutors, not those harmed by pornography.

Prostituted people in Sweden report having a bargaining advantage over their customers, who in turn know they can be reported for mistreating the person they purchased. This is something not found in countries like Germany and New Zealand, where prostitution is legal. The Swedish substantive equality prostitution law has been successful in reducing prostitution, and the exit programs do provide support for prostitutes to leave the “business” (Waltman, 2021, p.371). The apparent multiple disadvantages and problems that arise in seeking to address the intersections between prostitution and pornography laws are inadequate to the task of representing the perspectives and interests of those most directly harmed.

An alternative approach to criminal law is a civil rights strategy to view pornography as sex discrimination, originally conceived by Catharine MacKinnon and Andrea Dworkin, attempted in several U.S. jurisdictions. From this perspective, the ability to use and apply the law is accorded to those harmed, who are able to sue for damages either in a court or in an alternative administrative body. This civil rights approach to pornography was built upon the experiences and interests of those most harmed. In contrast to obscenity laws, criminal pornography law, or civil law approaches like sexual harassment, the ordinances based on civil rights strategies were drafted as a means of promoting substantive equality from the perspectives of the “injured persons”. The ordinances specifically address such intersectional problems as poverty, racial discrimination, childhood abuse, and the lack of alternative sources of income. Civil rights ordinances, more than any other kind of law, promote the interests of those most affected by the harms of pornography. They would, for the first time, give those harmed a voice in the legal process and thereby incentive public testimony of the injuries caused by pornography. No such incentive exists today.

Waltman asks for a shift in legal authority away from the state to make it more accessible to members of disadvantaged groups and their legitimate representatives whose commitment to achieving redress is bound to be stronger. There are other social maladies, such as hate crimes, systemic discrimination, and other forms of gender-based violence such as rape and domestic abuse, that would benefit from a similar change in focus toward the provision of civil rights. Waltman (2021, p.398) suggests an overall change in emphasis toward social groups, proposing that support for autonomous organizations and litigation would be a more appropriate approach for the twenty-first century. However, these arguments do not have the same weight in a Swedish context, where the government is regarded as a protector of vulnerable groups.

Climate politics show the same issues by an arguably unjust requirement that a consensus be reached among unequally situated parties, those who dominate and those who subordinate. This represents similar democratic problems to the fight against pornography. Both represent complex challenges to strong “vested interest” parties in society that contribute to the existing norms and reproduce harmful practices at the expense of those harmed. According to Waltman, there are aspects of global warming that, on a global scale, are related to social dominance and affect disadvantaged groups that will necessitate concerted legal challenges in the future (Waltman, 2021, p.399). Climate change will affect populations around the globe asymmetrically and unequally, which means developing nations will demand compensation from industrial countries who benefitted from cheap natural resources and the emission of greenhouse gases. The problems affecting global warming are, as with pornography, governed by a deliberative politics of consensus that is controlled by dominant nations and interests removed from those most adversely affected (Raymond et al., 2014).^3^

The most salient implication of the study that Waltman underscores is the intractable problems of inequality (Waltman, 2021, p.401). Should the issue be in the hands of civil society or should it belong to the state? The problem is somewhat of a dilemma, which Waltman illuminates through a huge number of empirical examples commented upon throughout the book. The knowledge he exhibits into the different politico-legal systems of the United States, Canada, and Sweden is impressive.

Waltman's position is that pornography and global warming can successfully—though on different levels—be countered only by placing the perspectives and interests of those most immediately affected at the law's center, which has not been the case over the years. The best way to achieve this is, according to Waltman, a legal design of the civil rights anti-pornography approach, which is consistent with the empowerment of organizations like women-led non-governmental organizations representing and assisting individuals who would have difficulties attempting to enforce legal rights on their own. This strategy represents a private strategy. It requires mobilizing both human and economic resources. By contrast, criminal law is in the hands of governmental representatives, not those who are victimized. The question of resources becomes then a democratic issue. Waltman's point is that if the governments do not act to apply their laws against pornography, there are no means for those affected to intervene. The civil right law strategy would shift the distribution of power and put the legal initiative into the hands of those who are hurt and who have the most substantial incentives to act.

## Author contributions

The author confirms being the sole contributor of this work and has approved it for publication.

## Footnotes

^1^Shapiros approach is distinct from one which the problem analyzed is a mere artifact of the theories and methods that are deployed to study it.

^2^However, prostitution as such seems not to have decreased. Prostitution has simply moved from the streets to the internet.

^3^See Raymond et al. (2014), analyzing challenges to climate change and violence against women.
